# Pregnancy-specific Reference Intervals for TSH and FT4: Novel Insights From Preconception-gestation Longitudinal Data

**DOI:** 10.1210/clinem/dgag082

**Published:** 2026-02-28

**Authors:** Leonie T L Warringa, Joris A J Osinga, Arash Derakhshan, Vincent W V Jaddoe, Sjoerd A A van den Berg, Robin P Peeters, Tim I M Korevaar

**Affiliations:** Academic Center for Thyroid Diseases, Department of Internal Medicine, Erasmus University Medical Center, 3000 CA Rotterdam, The Netherlands; The Generation R Study Group, Erasmus University Medical Center, 3000 CA Rotterdam, The Netherlands; Academic Center for Thyroid Diseases, Department of Internal Medicine, Erasmus University Medical Center, 3000 CA Rotterdam, The Netherlands; The Generation R Study Group, Erasmus University Medical Center, 3000 CA Rotterdam, The Netherlands; Academic Center for Thyroid Diseases, Department of Internal Medicine, Erasmus University Medical Center, 3000 CA Rotterdam, The Netherlands; The Generation R Study Group, Erasmus University Medical Center, 3000 CA Rotterdam, The Netherlands; The Generation R Study Group, Erasmus University Medical Center, 3000 CA Rotterdam, The Netherlands; Department of Paediatrics, Erasmus University Medical Center, 3000 CA Rotterdam, The Netherlands; Department of Clinical Chemistry, Erasmus University Medical Center, 3000 CA Rotterdam, The Netherlands; Academic Center for Thyroid Diseases, Department of Internal Medicine, Erasmus University Medical Center, 3000 CA Rotterdam, The Netherlands; The Generation R Study Group, Erasmus University Medical Center, 3000 CA Rotterdam, The Netherlands; Academic Center for Thyroid Diseases, Department of Internal Medicine, Erasmus University Medical Center, 3000 CA Rotterdam, The Netherlands; The Generation R Study Group, Erasmus University Medical Center, 3000 CA Rotterdam, The Netherlands; Division of Vascular Medicine and Pharmacology, Department of Internal Medicine, Erasmus University Medical Center, 3000 CA Rotterdam, The Netherlands

**Keywords:** thyroid function tests, reference values, preconception, pregnancy, thyrotropin, thyroxine

## Abstract

**Background:**

Pregnancy-specific changes in thyroid physiology have prompted the use of pregnancy-specific reference intervals to diagnose thyroid disease. However, such reference intervals are not widely available, and there is no evidence of their superiority over nonpregnancy reference intervals. Preconception data are understudied benchmarks to compare pregnancy-specific and nonpregnancy reference intervals. Moreover, the added value of free T3 (FT3) measurements, for example in cases of (subclinical) hyperthyroidism, remains to be quantified.

**Methods:**

This study was embedded within Generation R Next, a population-based prospective cohort from preconception through postpartum in Rotterdam. Prevalence of thyroid disease entities was assessed using both pregnancy-specific and nonpregnancy reference intervals during pregnancy and using nonpregnancy reference intervals in the preconception period. FT3 concentrations of both euthyroid participants and those with thyroid disease entities were compared in the preconception period and during pregnancy.

**Results:**

The study population included 1058 women during preconception and 2084 women during pregnancy. The prevalence of subclinical hypothyroidism during pregnancy was 1.5% with the use of nonpregnancy reference intervals and 3.6% with pregnancy-specific reference intervals vs 2.2% during preconception. The prevalence of subclinical hyperthyroidism during pregnancy was 11.1% with the use of nonpregnancy reference intervals and 1.5% with pregnancy-specific reference intervals vs 2.0% during preconception. Additional FT3 measurements reclassified 5.6% of subclinical hyperthyroidism cases to hyperthyroidism during preconception and 14.3% during pregnancy.

**Conclusion:**

This is the first study to assess the prevalence of (sub)clinical thyroid disease during pregnancy comparing nonpregnancy and pregnancy-specific reference intervals, while also comparing these prevalences to preconception data from the same source population. We show that pregnancy-specific reference intervals likely result in overdiagnosis of subclinical hypothyroidism and that FT3 has limited value in diagnosing (sub)clinical thyroid disease during pregnancy.

Correctly diagnosing thyroid dysfunction during pregnancy is important because untreated hypothyroidism is associated with a higher risk of adverse pregnancy outcomes (1-7). However, the diagnosis of thyroid disease during pregnancy is complicated by changes in thyroid physiology, such as changes in binding proteins, increased iodine clearance, placental degradation of T4 by deiodinase type 3, and stimulation of the TSH receptor by human chorionic gonadotropin (hCG). Generally, this results in a transient increase in free T4 (FT4) and a decrease in TSH concentrations at the end of the first trimester (8, 9). Pregnancy-specific changes in thyroid physiology have prompted the use of pregnancy-specific reference intervals to diagnose thyroid disease. Although pregnancy-specific reference intervals are considered the gold standard, most hospitals worldwide do not have such reference intervals available (10, 11). Moreover, there is hardly any evidence to support the use of pregnancy-specific reference intervals over reference intervals used for the general population (12). Most of our knowledge on this topic has come from studies solely assessing pregnant women. For example, in an individual participant data meta-analysis of 25 prospective cohort studies, it was shown that the use of trimester and pregnancy-specific reference intervals approximately triples the number of women diagnosed with overt or subclinical hypothyroidism as compared to nonpregnancy reference intervals (13). However, it remains challenging to establish the most accurate method for identifying true thyroid disease during pregnancy, primarily due to the lack of an appropriate benchmark for comparison. Previous observational studies have typically involved 2 separate cohorts from the same source population, which may introduce selection bias based on pregnancy status. In contrast, this study is the first to prospectively follow a cohort of nonpregnant women with a child wish, through conception and beyond, with consistent data collection throughout. This study design offers a robust framework for comparing nonpregnant reference intervals with pregnancy-specific reference intervals across both preconception and gestational periods. Furthermore, it provides a valuable benchmark for future research, particularly in cohorts with longitudinal measurements from preconception to pregnancy, enabling the exploration of individual thyroid function trajectories and their associations with pregnancy outcomes.

The diagnoses of thyroid dysfunction during pregnancy are predominantly based on TSH and FT4, while the added value of measuring free T3 (FT3) remains poorly quantified. Although TSH represents a more sensitive and long-term reflection of thyroid hormone availability as compared to FT4 and FT3, an additional FT3 measurement may provide further information in cases of (subclinical) hyperthyroidism (14-16). This potential of FT3 measurements has not been assessed during preconception or during pregnancy.

The aims of this study were to compare the prevalence of thyroid (dys)function as diagnosed by either nonpregnancy or pregnancy-specific TSH and FT4 reference intervals, to study this within a preconception and gestational subgroup from the same population, and to explore the added diagnostic value of FT3 during preconception and pregnancy.

## Materials and Methods

### Design and Study Population

This study was embedded within the Generation R Next study, a population-based prospective cohort from preconception onward in Rotterdam, the Netherlands, and is part of the Generation R Study (https://generationr.nl/next/) (17). The study was designed to identify preconception and early-pregnancy determinants of fertility, embryonic development, and childhood outcomes. Women and their partners residing in the Rotterdam area who were either planning pregnancy (within a year) or were pregnant (preferably less than 11 weeks) between August 2017 and July 2021 were eligible for inclusion. Women could enroll multiple times within the inclusion period [Supplemental Methods 1 (18)]. We included women with a blood sample available for measurement of TSH, FT4, FT3, and thyroid peroxidase antibodies (TPOAbs). We excluded women with known thyroid disease, thyroid-interfering medication usage, twin pregnancies, and/or pregnancies resulting from in vitro fertilization treatment. Participating women and partners provided written informed consent for participation, and the study was approved by the medical ethical committee of the Erasmus University Medical Center, Rotterdam, the Netherlands (2016-12-06, MEC2016-589).

### Thyroid Function Measurements

Serum samples were obtained during the first preconception and/or gestational visit. After sampling, plain tubes were centrifuged within 3 hours and serum was stored at −80 C. TSH, FT4, FT3, and TPOAbs were analyzed retrospectively using the same Lumipulse G automated chemiluminescent immunoassay system (Fujirebio). The Lumipulse assay did not show significant measurement deviations during pregnancy compared to outside pregnancy in a recent validation study (19). Of note, TSH and FT4 results were recalibrated according to recommendations for harmonization of TSH (20) and standardization of FT4 (21), respectively. Harmonization of TSH was assessed by external quality assessment (Stichting Kwaliteitsbewaking Medische Laboratoriumdiagnostiek). Standardization of FT4 was assessed by direct comparison to the reference measurement procedure (Ref4U, University of Ghent).

The day-to-day coefficients of variation were 2.2% for TSH, 2.1% for FT4, and 2.1% for FT3 at an average target of 1.46 mU/L, 14.3 pmol/L, and 3.64 pmol/L, respectively. The intra-assay coefficients of variation were 2.7% for TSH, 2.1% for FT4, and 2.6% for FT3 at an average target of 2.78 mU/L, 17.8 pmol/L, and 6.58 pmol/L, respectively. For TPOAb, there was an overall precision of ≤6.8% (combination of within-run, between-run, and between-day precision data), and TPOAb positivity was defined according to the assay manufacturer’s cutoff of > 6.9 IU/mL. Nonpregnancy reference intervals for TSH, FT4, and FT3 were those provided by the assay manufacturer and were as follows: 0.56 to 4.27 mU/L for TSH, 13.5 to 24.3 pmol/L for FT4, and 3 to 5.7 pmol/L for FT3.

### Statistical Analysis

Pregnancy-specific reference intervals were defined in accordance with the American Thyroid Association Guidelines as the 2.5th to 97.5th percentiles in TPOAb-negative women (12). In addition, for sensitivity analyses we also calculated 2.5th to 97.5th percentiles reference intervals in the preconception study population. Thyroid disease entities (eg, subclinical hypothyroidism) were defined based on lower and upper limits of the applicable reference interval [Supplemental Methods 2 (18)]. Differences in median concentrations of thyroid hormone across thyroid disease entities were assessed by linear regression of log-transformed thyroid hormone against thyroid disease entities with euthyroidism as a reference category. Between subgroups differences were assessed with a 2-sample *t*-test, Wilcoxon signed-rank test, and/or 1-way ANOVA for continuous measurements depending on the distribution and number of subgroups. For categorical measurements, chi-square statistics were used. All analyses were performed using R statistical software version 4.1.2 [package “ggplot2,” “dplyr,” and “ggsankey” (22-25)].

## Results

**Figure 1:**
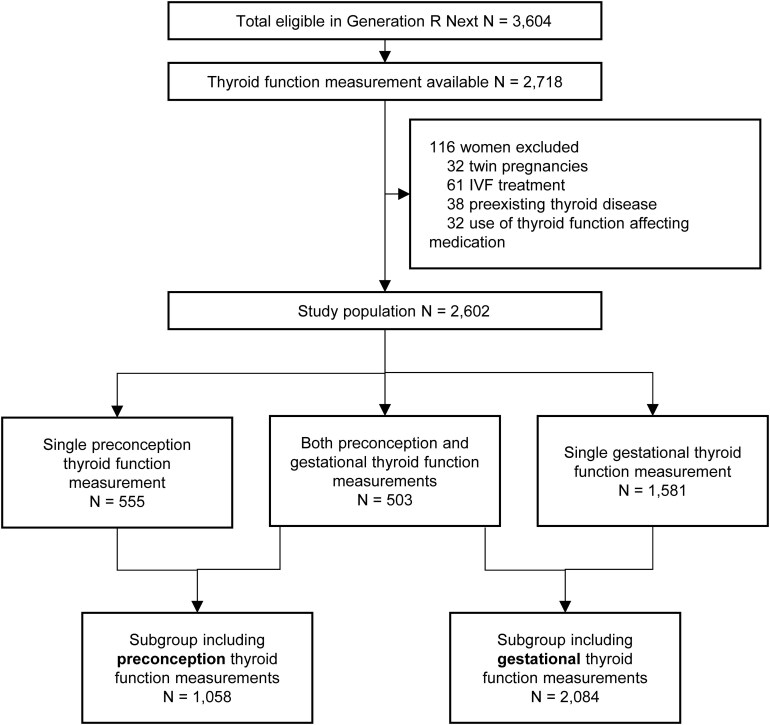
Flowchart.

After exclusions, the final study population comprised 2602 women, with a total of 1058 preconception and 2084 gestational thyroid function measurements (n = 555 only preconception, n = 1581 only gestational, and n = 503 both preconception and gestational thyroid function measurements; Fig. 1). Median time to pregnancy was 4.2 months [95% range 0.9-13.0, Table S1 (18)]; during preconception the median TSH was 1.67 mU/L (95% range 0.61-4.57), mean FT4 was 18 pmol/L (95% range 13.4-23.1), and mean FT3 was 4.7 pmol/L (95% range 3.8-5.8). Mean gestational age at time of gestational thyroid function measurements was 8.9 weeks (95% range 6-13.1; Table 1), and during pregnancy the median TSH was 1.35 mU/L (95% range 0.14-3.88), mean FT4 was 17.7 pmol/L (95% range 13.2-23.6), and mean FT3 was 4.8 pmol/L (95% range 3.9-5.9). There was a small difference in gestational TSH between the group with only gestational thyroid function measurement and the group with both preconception and gestational thyroid function measurement but no other differences in key variables [Table S1 (18)].

**Table 1:** Baseline characteristics of the study population

	Preconception	Gestational
n	1058	2084
Age at blood sampling (years)	32 (24-42)	32 (22-40)
Gestational age at blood sampling (weeks)	NA	8.9 (6-13.1)
Parity at intake, n (%)		
0	730 (76.5)	1202 (66.2)
1	173 (18.1)	454 (25)
≥2	51 (5.3)	161 (8.9)
BMI (kg/m^2^) before pregnancy	25.2 (18.5-38.9)	24.2 (18.4-35.9)
Ethnicity, n (%)		
Dutch	600 (60.8)	1214 (64.3)
Surinamese	92 (9.3)	114 (6)
European	60 (6.1)	116 (6.1)
Dutch Antilles	47 (4.8)	82 (4.3)
Other Western	45 (4.6)	75 (4)
Other non-Western	143 (14.5)	287 (15.2)
Education, n (%)		
Low	8 (0.8)	19 (1)
Medium	212 (21.7)	524 (28)
High	757 (77.5)	1330 (71)
Smoking, n (%)		
Never	531 (60.7)	965 (54.3)
Former smoker	265 (30.3)	529 (29.8)
Active smoker	79 (9)	284 (16)
Thyroid function test		
TSH (mU/L), median (95% range)	1.67 (0.61-4.57)	1.35 (0.14-3.88)
FT4 (pmol/L)	18 (13.4-23.1)	17.7 (13.2-23.6)
FT3 (pmol/L)	4.7 (3.8-5.8)	4.8 (3.9-5.9)
TPOAb positivity, n (%)	109 (10.5)	225 (10.8)

Mean (95% range) is presented, unless otherwise specified. Overlap of 37 women (1.7% of total sample), which are presented in the preconception and gestational group but in different enrollments. Number of missing: parity = 357, gestational age = 1, BMI = 280, ethnicity = 259, education = 277, smoking = 426, TSH = 2, FT4 = 11, FT3 = 7, TPOAb positivity = 23.

Abbreviations: BMI, body mass index; FT3, free T3; FT4, free T4; TPOAb, thyroid peroxidase antibodies.

### Reference Intervals and Prevalence of Thyroid Disease

The calculated reference intervals for TSH were 0.15 to 3.44 mU/L in pregnancy (ie, pregnancy-specific reference interval; Table 2) and 0.62 to 3.88 mU/L during the preconception period [Table S2A (18)]. The prevalence of subclinical hyperthyroidism during pregnancy was 11.1% with the use of nonpregnancy reference intervals and 1.5% with pregnancy-specific reference intervals vs 2.0% during preconception (Table 3). The prevalence of isolated hypothyroxinemia during pregnancy was 2.8% with the use of nonpregnancy reference intervals and 2.3% with pregnancy-specific reference intervals vs 2.4% during preconception (Table 3). The prevalence of subclinical hypothyroidism during pregnancy was 1.5% with the use of nonpregnancy reference intervals and 3.6% with pregnancy-specific reference intervals vs 2.2% during preconception (Table 3). Prevalences were similar in the subgroup of women with both preconception and gestational thyroid function measurements [Table S2C (18)].

**Table 2:** Reference intervals

	Provided by manufacturer	Calculated 2.5th-97.5th percentile
	Nonpregnancy	Pregnancy-specific
**TSH (mU/L)**	0.56-4.27	0.15-3.44
FT4 (pmol/L)	13.5-24.3	13.4-23.3
FT3 (pmol/L)	3-5.7	3.9-5.9

Abbreviations: FT3, free T3; FT4, free T4.

**Table 3:** Prevalence of thyroid (dys)function of women with only preconception thyroid function test (n = 555) and only gestational thyroid function test (n = 1581)

	Preconception	Gestational	
Reference interval	Nonpregnancy (%)	Nonpregnancy (%)	Pregnancy-specific (%)
n	550	1579	1579
Euthyroidism	503 (91.5)	1303 (82.5)	1417 (89.7)
Hypothyroidism	4 (0.7)	3 (0.2)	4 (0.3)
Hypothyroidism TPO+	2 (0.4)	3 (0.2)	4 (0.3)
Subclinical hypothyroidism	12 (2.2)	23 (1.5)	57 (3.6)
Subclinical hypothyroidism TPO+	4 (0.7)	14 (0.9)	26 (1.7)
Hypothyroxinemia	13 (2.4)	44 (2.8)	36 (2.3)
Hyperthyroxinemia	7 (1.3)	3 (0.2)	18 (1.1)
Subclinical hyperthyroidism	11 (2)	175 (11.1)	23 (1.5)
Hyperthyroidism	0	27 (1.7)	24 (1.5)

Missing in TSH or free T4: n = 5 for preconception group, n = 2 for gestational group.

Abbreviation: TPO, thyroid peroxidase.

**Figure 2:**
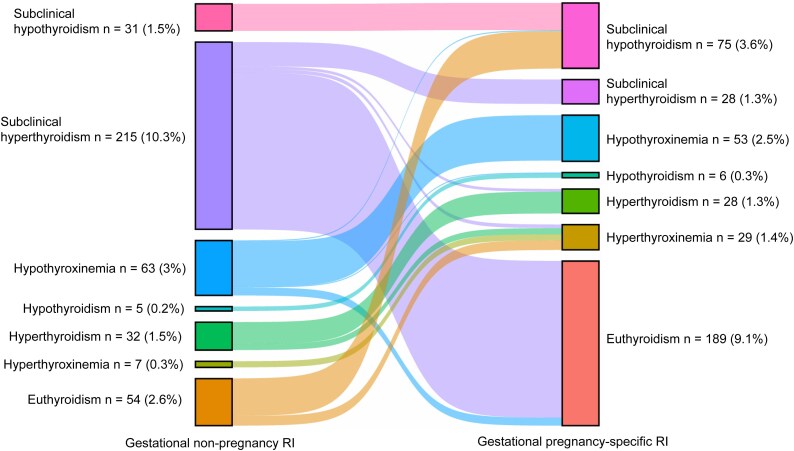
Reclassification of thyroid disease entities according to nonpregnancy- and pregnancy-specific RIs during pregnancy (n = 2084). Reclassification from euthyroidism to euthyroidism [n = 1671 (80.2%)] not shown. Abbreviation: RI, reference interval.

Figure 2 shows the reclassification of different thyroid disease entities and euthyroidism during pregnancy between nonpregnancy reference intervals (left) and pregnancy-specific reference intervals (right). With the use of pregnancy-specific reference intervals, 180 out of 215 (84%) with subclinical hyperthyroidism were reclassified to euthyroid. At the same time, however, 43 out of 1725 (3%) of euthyroid women were reclassified to subclinical hypothyroidism. In comparison, if we were to apply the same concept of calculated reference intervals to the preconception study population, similar but smaller differences in prevalence of subclinical hypothyroidism (2.6-3.3%) and isolated hypothyroxinemia (2.1-2.5%) would occur [Fig. S1 and Table S2B (18)]. No disease entities would be reclassified to euthyroid when using calculated 2.5th to 97.5th percentiles reference intervals among preconception women.

### Diagnostic Value of FT3

In euthyroid women, the median FT3 was the same during preconception as during pregnancy (median [95% range]: 4.7 [3.8-5.7] pmol/L vs 4.7 [3.9-5.8] pmol/L, respectively; Table 4). Hyperthyroidism was associated with a higher FT3 during preconception and during pregnancy (median [95% range]: 11.2 [7.1-15.3] pmol/L vs 6.1 [4.9-13.1] pmol/L, *P* < .01), but subclinical hyperthyroidism was only associated with a higher FT3 during pregnancy. Additional FT3 measurement reclassified 5.6% of subclinical hyperthyroidism cases to hyperthyroidism during preconception and 14.3% during pregnancy [and 7.9% with the use of nonpregnancy reference intervals during pregnancy, Table S3 (18)]. Isolated hypothyroxinemia was associated with a lower median FT3 in both the preconception and gestation groups (median [95% range]: 4.5 [3.2-5.7] pmol/L and 4.5 [3.6-5.7] pmol/L, *P* < .01).

**Table 4:** FT3 concentrations in preconception and gestational thyroid disease entities

	Preconception FT3 Nonpregnancy reference interval					Gestational FT3 Pregnancy-specific reference interval				
	n	Low n (%)	High n (%)	Median (95% range)	*P*	n	Low n (%)	High n (%)	Median (95% range)	*P*
Euthyroidism	965	0	27 (2.8)	4.7 (3.8-5.7)	Ref	1860	38 (2.0)	35 (1.9)	4.7 (3.9-5.8)	Ref
Hypothyroidism	5	0	0	4.2 (3.9-4.3)	<.01	6	0	0	4.5 (4.2-4.7)	.20
Hypothyroidism TPO+	3	0	0	4.2 (3.9-4.3)		5	0	0	4.5 (4.2-4.5)	.16
Subclinical hypothyroidism	28	0	4 (14.3)	4.8 (3.8-6.3)	.44	75	3 (4)	4 (5.3)	4.8 (3.8-6.2)	.04
Subclinical hypothyroidism TPO+	9	0	1 (11.1)	4.7 (3.8-5.7)	.75	30	3 (10)	3 (10)	4.6 (3.7-6.2)	.55
Hypothyroxinemia	22	0	1 (4.5)	4.5 (3.2-5.7)	<.01	53	6 (11.3)	0	4.5 (3.6-5.7)	<.01
Hyperthyroxinemia	10	0	1 (10)	5.3 (4.4-6.1)	<.01	29	0	0	5.1 (4-5.8)	<.01
Subclinical hyperthyroidism	18	0	1 (5.6)	4.8 (4-6.1)	.45	28	0	4 (14.3)	5.2 (4.1-6.2)	<.01
Hyperthyroidism	2	0	2 (100)	11.2 (7.1-15.3)		28	0	16 (57.1)	6.1 (4.9-13.1)	<.01

Preconception thyroid disease entities based on manufacturer assay cutoff; gestational thyroid disease entities based on calculated 2.5th to 97.5th percentile pregnancy-specific reference interval; *P*-value derived from linear regression of log transformed FT3 with “euthyroidism” as reference category; no *P*-value presented for categories with <5 subjects. The 95% range is the 2.5th to 97.5th percentile.

Abbreviations: FT3, free T3; Ref, reference category; TPO, thyroid peroxidase.

## Discussion

We demonstrated that a pregnancy-specific reference interval considerably decreases the prevalence of subclinical hyperthyroidism and increases the prevalence of subclinical hypothyroidism but has no relevant impact on the prevalence of isolated hypothyroxinemia. Although 7.9% to 14.3% of cases of gestational subclinical hyperthyroidism could be reclassified when a FT3 measurement is added, the added diagnostic value of FT3 in both preconception and pregnancy remains very limited owing to the small number of women and lack of clinical consequences of the reclassification.

Current guidelines recommend the use of pregnancy-specific TSH and FT4 reference intervals for each trimester to diagnose thyroid disease to account for gestational thyroid physiology changes (12). However, such recommendations are based on little evidence, and the lack of studies examining the true effects of pregnancy-specific reference intervals on thyroid disease prevalence has never been validated with preconception data as a reference. We show that, on 1 hand, pregnancy-specific reference intervals correct for the physiological thyroidal stimulation during pregnancy by normalizing the increase of subclinical hyperthyroidism. On the other hand, our results strongly indicate that pregnancy-specific TSH and FT4 intervals lead to an increase in the prevalence of subclinical hypothyroidism. Unfortunately, we lacked the power to adequately study overt hypothyroidism. Using nonpregnancy reference intervals, the prevalence of subclinical hypothyroidism was 2.2% during preconception and decreased to 1.5% during pregnancy (in the repeated measurements subset from 3.2% to 1.6%). This decrease in prevalence is in line with the known decrease in TSH due to additional thyroidal stimulation by hCG during pregnancy. However, completely opposite to normal physiology, with pregnancy-specific reference intervals the subclinical hypothyroidism prevalence during pregnancy increases to 3.6%. This difference seems to be predominantly driven by the TPOAb-negative subclinical hypothyroidism group—a group where normal thyroidal stimulation by hCG can be expected, which results in a lower calculated upper limit of TSH (26, 27). In addition, there were no similar trends identified for overt hypothyroidism. Altogether, these data indicate that we should question whether the women who are diagnosed with subclinical hypothyroidism by pregnancy-specific reference intervals but not by reference intervals used for the general population (ie, nonpregnancy) truly have subclinical hypothyroidism or are misdiagnosed. Interestingly, the concept of overdiagnosis when using calculated TSH and FT4 reference intervals is also seen in the preconception group, with an increase in the prevalence of subclinical hypothyroidism by 50%. Although it concerns small numbers, a few high TSH levels might be caused by macro TSH, regardless of the reference interval used (28, 29). The discussion about subclinical hypothyroidism outside of pregnancy is rightfully much more focused on overdiagnosis than underdiagnosis. Moreover, outside of pregnancy, population-based reference interval determinations are not a point of discussion. Before clinical guidelines can change recommendations on the use of pregnancy-specific intervals, the most important knowledge gap that needs to be filled is whether or not the group of women that is additionally diagnosed with subclinical hypothyroidism due to pregnancy-specific reference intervals has a higher risk of adverse pregnancy outcomes.

Using nonpregnancy reference intervals during pregnancy would have 2 consequences, namely (1) a relevant increase in the prevalence of subclinical hyperthyroidism and (2) a small increase in the prevalence of isolated hypothyroxinemia. The vast majority of the cases of subclinical hyperthyroidism during pregnancy represent transient, hCG-mediated gestational hyperthyroidism, a physiological condition. The incidence of Graves' disease during pregnancy is low (approximately .05%), and Graves' disease during pregnancy typically has a milder course (30).

Therefore, besides intensifying clinical monitoring, neither of these changes would have other clinical or therapeutic consequences (12).

Interestingly, the prevalence of isolated hypothyroxinemia outside of pregnancy was much more similar to that during pregnancy in our study than what is typically expected. This might suggest that isolated hypothyroxinemia is not as pregnancy-specific as is hypothesized (2, 8, 31). On the other hand, in a recent English study, the prevalence of isolated hypothyroxinemia in women planning pregnancy was >10-fold lower than in the current study (2.4% vs 0.2%) (32). In that study, different assays were used across the UK, but fixed TSH and FT4 limits were used to define thyroid disease entities. Therefore, this difference could be an artifact of different assay and cutoff uses, as supported by the interassay differences studied by the International Federation of Clinical Chemistry and Laboratory Medicine Committee for Standardization of Thyroid Function Tests (21). Another explanation might be that our assay underestimates the FT4 concentration, as our assay has a median deviation of −5.4 to −5.8 pmol/L for FT4 after recalibration (21). Further insights will be gained when subsequent studies investigate the individual course of thyroid function from preconception to gestation within the same women measured with the same analyzer.

Assessment of (F)T3 in addition to TSH and FT4 is more common practice for diagnosing hyperthyroidism outside of pregnancy than during pregnancy. This is because hyperthyroidism during pregnancy is mostly physiological and thus hardly ever a treatment indication (except in Graves' disease or toxic nodule/goiter) and because total T3 measurements are mostly available but remain difficult to interpret during pregnancy due to the rise in thyroxine binding globulin. This is the first large study to assess the diagnostic value of FT3 during pregnancy, and we were also able to compare diagnostic consequences with preconception data from the same source population. We identified that FT3 is lower in overt hypothyroidism and higher in overt hyperthyroidism as expected. Interestingly, median FT3 was lower in hypothyroxinemia both before and during pregnancy. Although the Netherlands has a known adequate iodine status, we lack individual data on iodine status to study iodine deficiency as a possible underlying cause of isolated hypothyroxinemia. With nonpregnancy reference intervals, none of the women with thyroid function test abnormalities had a FT3 below the 2.5th percentile cutoff in either preconception or pregnancy, indicating that low FT3 has no diagnostic value. Furthermore, we also identified that in women with subclinical hyperthyroidism prior to pregnancy, the FT3 concentrations are similar to euthyroid women, but for subclinical hyperthyroidism during pregnancy the FT3 concentrations were higher than in euthyroid women. This fits with the expected pattern of TSH receptor stimulation by hCG during pregnancy. Interestingly, assessment of FT3 would change the diagnosis to overt hyperthyroidism for 7.9% to 14.3% of the subclinical hyperthyroidism group, depending on the reference interval used. However, this reclassification would not have clinical implications beyond the need for intensified monitoring. There is no indication for antithyroid drug treatment in cases of overt hyperthyroidism during pregnancy unless it is caused by Graves' disease or nodular thyroid disease, given that transient gestational hyperthyroidism, a physiological condition, is typically self-limiting (12). Thus, our data indicate that the added (diagnostic) value of a FT3 measurement in pregnancy is limited and should only be considered when there is a suspicion of Graves' disease or a T3-producing nodule.

In this unique study, we were able to study the diagnostic consequences of applying different thyroid function reference intervals and the role of FT3 during pregnancy, with a direct comparison to a large cohort of women from the same source population assessed during the preconception period. This provides a novel and more robust benchmark compared to previous pregnancy-focused studies, which included 2 cohorts from the same source population, in contrast to the single cohort in the current study (10, 33-35).

Additionally, data collection, including sample handling and measurement, was done in a consistent manner in both preconception and gestation, largely eliminating intra-assay and intralaboratory variations in TSH and FT4 measurements (36). This consistency enhances the reliability of our findings and provides a clearer understanding of the true impact of different reference intervals.

In this study, there were almost twice as many gestational thyroid function tests than preconception thyroid function tests available, which highlights the challenges of recruiting participants during the preconception period. The difficulty of recruiting women during their preconception period renders this dataset susceptible to differential participation across the different phases (preconception and pregnancy). However, there was only a small difference in median TSH between women who participated only during pregnancy and women who participated in both phases. Another restriction of this study is the absence of total T3 measurements, which are preferred over FT3 measurements by some but not all specialists, because assays estimating FT3 are generally considered less robust than those for total T3 and FT4 (37). Nonetheless, the coefficients of variation for FT3 in our assay were comparable to those of TSH and FT4. Moreover, all FT3 measurements conducted during both preconception and pregnancy were analyzed using the same assay, ensuring their comparability between these 2 periods.

In conclusion, this is the first study to assess the prevalence of (sub)clinical thyroid disease during pregnancy comparing nonpregnancy and pregnancy-specific reference intervals and comparing these prevalences with preconception data from the same source population. We showed that we should question the relevance of using pregnancy-specific reference intervals because it results in an increased prevalence of subclinical hypothyroidism; it is unclear if this additionally diagnosed group also faces an increased risk of adverse (pregnancy) outcomes. Moreover, the lack of treatment benefit in this group and the limited additional value of diagnosing gestational hypothyroxinemia argues against the use of pregnancy-specific reference intervals.

In addition, our data indicated that FT3 is of limited value in diagnosing (sub)clinical thyroid disease, other than overt hyperthyroidism, in both preconception and pregnancy. Future research involving repeated thyroid function measurements in both the preconception and gestational periods could help clarify whether thyroid dysfunction during pregnancy is preexisting and assess individual trajectories from preconception to pregnancy for further insights. Nonetheless, this study has already demonstrated the importance of preconception thyroid function measurements to enhance the evaluation of gestational thyroid function measurements in research.

## Other Data Collection

Data collection during visits included questionnaires, anthropometry (eg, weight), blood and urine samples, interviews, optometry, body composition and bone density measurements, and a (doppler) ultrasound of the fetus in the case of pregnancy.

## Data Availability

Some or all datasets generated during and/or analyzed during the current study are not publicly available but are available from the corresponding author on reasonable request.
